# Missed Opportunities for Diagnosis and Treatment of Diabetes, Hypertension, and Hypercholesterolemia in a Mexican American Population, Cameron County Hispanic Cohort, 2003–2008

**DOI:** 10.5888/pcd9.110298

**Published:** 2012-08-02

**Authors:** Susan P. Fisher-Hoch, Kristina P. Vatcheva, Susan T. Laing, M. Monir Hossain, M. Hossein Rahbar, Craig L. Hanis, H. Shelton Brown, Anne R. Rentfro, Belinda M. Reininger, Joseph B. McCormick

**Affiliations:** Author Affiliations: Kristina P. Vatcheva, Belinda M. Reininger, Joseph B. McCormick, University of Texas School of Public Health, Brownsville, Texas; Susan T. Laing, M. Monir Hossain, M. Hossein Rahbar, Craig L. Hanis, H. Shelton Brown III, University of Texas Health Science Center-Houston, Houston, Texas; Anne R. Rentfro, University of Texas at Brownsville, Brownsville, Texas.

## Abstract

**Introduction:**

Diabetes, hypertension, and hypercholesterolemia are common chronic diseases among Hispanics, a group projected to comprise 30% of the US population by 2050. Mexican Americans are the largest ethnically distinct subgroup among Hispanics. We assessed the prevalence of and risk factors for undiagnosed and untreated diabetes, hypertension, and hypercholesterolemia among Mexican Americans in Cameron County, Texas.

**Methods:**

We analyzed cross-sectional baseline data collected from 2003 to 2008 in the Cameron County Hispanic Cohort, a randomly selected, community-recruited cohort of 2,000 Mexican American adults aged 18 or older, to assess prevalence of diabetes, hypertension, and hypercholesterolemia; to assess the extent to which these diseases had been previously diagnosed based on self-report; and to determine whether participants who self-reported having these diseases were receiving treatment. We also assessed social and economic factors associated with prevalence, diagnosis, and treatment.

**Results:**

Approximately 70% of participants had 1 or more of the 3 chronic diseases studied. Of these, at least half had had 1 of these 3 diagnosed, and at least half of those who had had a disease diagnosed were not being treated. Having insurance coverage was positively associated with having the 3 diseases diagnosed and treated, as were higher income and education level.

**Conclusions:**

Although having insurance coverage is associated with receiving treatment, important social and cultural barriers remain. Failure to provide widespread preventive medicine at the primary care level will have costly consequences.

## Introduction

Hispanics are projected to comprise 30% of the US population by 2050 (1). Mexican Americans, the largest ethnically distinct subgroup among Hispanics, are at high risk for becoming overweight or obese, predisposing them to type 2 diabetes and metabolic and cardiovascular disease (2). These chronic diseases lead to substantial increases in disability and premature death (3,4). Identifying and addressing obstacles to the early prevention, diagnosis, and treatment of chronic diseases in this population could allow us to address disparities in disease burdens (4).

Approximately 50% of Mexican Americans residing on the United States border with Mexico are obese, compared with 39.3% of Mexican Americans nationally (2,4). In 2006, the American Diabetes Association estimated prevalence of diabetes among Mexican Americans living along the US border in Texas at 14.7%, considerably higher than the national prevalence among Mexican Americans (10.4%) and non-Hispanic whites (6.5%) (2,5-8). Overall age-adjusted prevalence of hypertension in the United States is about 30%. Blacks are most affected (42%); prevalence for non-Hispanic whites is 28.8% and for Mexican Americans, 25.5% (9). However, in the US-Mexico border region, age-adjusted hypertension is reportedly 47.6% (10). Elevated low-density lipoprotein (LDL) cholesterol in the United States affects about 33.5% of the population overall and 27.7% of Mexican Americans (11). These 3 chronic diseases adversely affect the local community, health care system, and economy.

The Cameron County Hispanic Cohort (CCHC), initiated in 2003, is an ongoing study of Mexican American participants from randomly selected households in Brownsville, Cameron County, Texas, on the US-Mexico border (8). This large cohort study documents sociodemographic, clinical, behavioral, and biologic characteristics of Cameron County Mexican Americans, one of the poorest communities in the United States (12). This community experiences severe disparities in income, education, and health care access (7,8) Ninety-two percent of Brownsville’s estimated population of 170,000 is Mexican American and have low high school graduation rates and low incomes (8,13). The objectives of our study were to use cross-sectional baseline data from the CCHC to determine the extent to which 3 chronic diseases — diabetes, hypertension, and hypercholesterolemia — are undiagnosed and untreated in this minority population with severe health disparities and what factors influence failure to diagnose and treat these diseases.

## Methods

We used data collected prospectively from CCHC participants from 2003 to 2008 (8). Participants were aged 18 years or older and were randomly selected from Mexican American households in Brownsville, Texas. The Committee for the Protection of Human Subjects at the University of Texas Health Science Center-Houston approved all protocols and consent forms.

We invited participants from randomly selected households to attend our Clinical Research Unit for an individual interview and examination. We obtained informed consent, conducted interviews and physical examinations, and obtained extensive data on socioeconomic and educational status and on personal and family medical histories.

We performed anthropometric measures as described previously, including height, weight, and waist circumference, and we calculated body mass index (BMI) (8). We used the Mini-Mental State Examination standard protocols (Psychological Assessment Resources, Inc, Lutz, Florida) to assess English and Spanish language literacy (14). We took 3 separate supine blood pressure readings, and we recorded heart rate.

We collected, stored, and processed blood specimens for measurements, including fasting blood glucose, fasting insulin, hemoglobin A1c (A1c), and blood chemistries (8). We used a Glucostat analyzer (Model 27, YSA, Inc, Yellow Springs, Ohio) to measure fasting blood glucose, enzyme-linked immunosorbent assays (Mercodia, Uppsala, Sweden) to measure blood insulin levels, and GLYCO-Tek Affinity Columns (Helena Laboratories, Beaumont, Texas) to measure A1c on frozen whole blood (15). We obtained fasting lipid and liver panels and high sensitivity C-reactive protein in a Clinical Laboratory Improvement Amendments-approved medical laboratory.

We categorized obesity as a BMI of 30 kg/m^2^ or greater. We used the 2010 definition of diabetes of the American Diabetes Association (ADA) and the World Health Organization, which includes an A1c of 6.5% or greater (16,17). We did not perform the oral glucose tolerance test because of time and cost constraints. We categorized participants as having diagnosed diabetes if they said they had been told by a health care provider that they had diabetes or if they were taking hypoglycemic medication; participants with undiagnosed diabetes were those who had not been told they had diabetes but whose laboratory data met the 2010 ADA criteria for diabetes. Similarly, we described participants as having diagnosed hypertension if they had been told by a health care provider that they had high blood pressure or if they were taking antihypertensive medication. Participants were classified as undiagnosed if a health care provider had not told them that they had high blood pressure or if they were not taking blood pressure-lowering medication but their mean systolic blood pressure was 140 mm Hg or higher or their mean diastolic blood pressure was 90 mm Hg or higher. Participants with diagnosed hypercholesterolemia were those who had been told by a health care provider that they had high cholesterol or those who were taking lipid-lowering medication. Participants were categorized as having undiagnosed hypercholesterolemia if they had a total fasting cholesterol of 200 mg/dL or greater but had not been told by a health care provider that they had high cholesterol or if they were not taking cholesterol-lowering medication. Participants with any form of private medical insurance, Medicaid, or Medicare were considered to have health insurance.

We asked participants what medications they were taking and reconciled what they reported against the actual medications they brought to the clinic. We subsequently checked approximately 7,500 individual medication entries against Mexican and American pharmacopeias and Internet searches and categorized medications by indication for diabetes, hypertension, or hypercholesterolemia. Diabetes medications included insulin or any oral hypoglycemic medication (ie, sulfonylureas, biguanides, thiazolidinediones, dipeptidyl peptidase, or α and β glucosidase inhibitors). Hypertension medications included a range of commonly used medications, principally diuretics, α- and β-blocking drugs, α-adrenergic agents, angiotensin-converting enzyme inhibitors, calcium channel blockers, and angiotensin II receptor antagonists. Lipid-lowering drugs included statins, fibrate-class drugs, niacin, omega-3 fatty acids, and cholestyramine. We included drugs with Spanish-language labels or formulations unavailable in the United States and assumed that they had been purchased in Mexico, where prescriptions are not required.

Data were weighted for analysis to correct for sampling bias based on census data to account for age, sex, census tract or block, and household clustering (8). We used student’s *t* test to compare means of groups for continuous variables and the χ^2^ test for categorical variables. We built 6 multivariable logistic regression models with outcomes of “undiagnosed” and “not receiving appropriate medication” for each of the 3 conditions. All independent variables that were significantly associated with the outcomes at significance level *P* < .10 were included in the models. Variables with no contribution to the fit of multivariable models were excluded. We report odds ratios and 95% confidence intervals. The level of significance for all tests was set at *P <* .05. The analyses used SAS 9.2 TS level 1MO (SAS Institute, Inc, Cary, North Carolina).

## Results

Among the 2,000 participants studied, the weighted prevalence of diabetes was 30.7%, hypertension, 30.5%, and hypercholesterolemia, 48.2%. The combined prevalence among participants of any 1 or more of the 3 chronic conditions, diagnosed or undiagnosed, was 69.6%. Approximately half of participants with diabetes (49.7%) or with hypercholesterolemia (51.3%) and 84% of participants with hypertension had had the condition diagnosed previously.

Several variables were associated with having undiagnosed (Table 1 and Table 2) and untreated (Table 3 and Table 4) diabetes, hypertension, and hypercholesterolemia. To further examine these associations we used a logistic regression model that controlled for confounders and interactions (Table 5). Younger participants were significantly less likely to have had all 3 conditions diagnosed and treated. Sex and country of birth were not associated with diagnosis or treatment, and an association between poverty and diagnosis could not be confirmed in the model.

**Table 1:** Univariable Analysis of Weighted Data for Continuous Variables for Diagnosis, Cameron County Hispanic Cohort (N = 2,000), 2000–2008

Variable	Diagnosed	Diabetes (n = 593)		Hypertension (n = 633)		Hypercholesterolemia (n = 958)	

		Mean (SE)	Mean Difference (95% CI)	Mean (SE)	Mean Difference (95% CI)	Mean (SE)	Mean Difference (95% CI)
Age in years	Yes	57.4 (1.1)	8.6 (4.7 to 12.6)	60.1 (0.9)	5.9 (1.2 to 10.7)	54.5 (1.1)	6.5 (2.9 to 10.1)
	No	48.8 (1.8)		54.1 (2.5)		48.0 (1.5)	
Household size	Yes	3.4 (1.2)	−0.12 (−0.51 to 0.27)	3.2 (0.1)	0.01 (−0.4 to 0.4)	3.4 (0.1)	−0.2 (−0.6 to 0.10)
	No	3.5 (0.2)		3.2 (0.2)		3.7 (0.1)	
Higher level of education	Yes	7.7 (0.3)	−1.99 (−2.9 to −1.1)	8.3 (0.3)	−0.5 (−1.7 to 0.6)	8.9 (0.3)	−1.4 (−2.2 to −0.6)
	No	9.7 (0.4)		8.8 (0.6)		10.3 (0.3)	
MMSE: Spanish test score^a^	Yes	30.6 (1.6)	−1.8 (−6.57 to 2.89)	32.4 (1.0)	1.0 (−7.6 to 9.5)	32.3 (1.2)	−1.8 (−5.2 to 1.6)
	No	32.4 (1.8)		31.4 (4.0)		34.1 (1.3)	
MMSE: English test score^a^	Yes	17.6 (1.6)	−7.69 (−12.5 to −2.9)	20.3 (1.4)	−2.2 (−7.6 to 3.3)	21.6 (1.3)	−4.6 (−8.2 to −1.0)
	No	25.3 (1.8)		22.4 (2.5)		26.2 (1.4)	
Years resident in Brownsville	Yes	30.9 (1.3)	4.2 (−0.3 to 8.6)	32.1 (1.4)	3.5 (−4.6 to -11.5)	28.2 (1.3)	3.4 (−1.5 to 8.2)
	No	26.7 (2.0)		28.7 (4.6)		24.8 (2.1)	

Abbreviations: MMSE, Mini-Mental State Examination; SE, standard error; CI, confidence interval.

^a^ English and Spanish literacies are assessed using the language panels from the MMSE package. Scores are from 0 to 45 points on each test (14).

**Table 2:** Univariable Analysis of Weighted Data for Categorical Variables for Diagnosis, Cameron County Hispanic Cohort (N = 2,000), 2000–2008

Categorical Variable	Diabetes (n = 593)		Hypertension (n = 633)		Hypercholesterolemia (n = 958)	

	Undiagnosed n/Total (%)^a^	OR (95% CI)^b^	Undiagnosed n/Total (%)^a^	OR (95% CI)^b^	Undiagnosed n/Total (%)^a^	OR (95% CI)^b^
Male	99/200 (54.6)	0.95 (0.62-1.44)	38/203 (17.0)	1.24 (0.72-2.15)	181/354 (50.9)	1.09 (0.76-1.57)
Female	198/392 (55.9)		60/431 (14.2)		290/614 (48.7)	1 [Reference]
Born in Mexico	208/405 (54.6)	0.94 (0.59-1.48)	65/409 (15.4)	1.07 (0.57-1.99)	323/656 (48.1)	0.81 (0.54-1.20)
Born in USA	86/183 (36.8)	1 [Reference]	30/216 (14.5)	1 [Reference]	145/300 (53.5)	1 [Reference]
Graduated from high school	111/190 (67.6)	2.19 (1.39-3.46)	39/197 (21.4)	1.89 (1.11-3.23)	221/403 (57.0)	1.65 (1.14-2.39)
Not graduated from high school	186/401 (48.8)	1 [Reference]	59/435 (12.6)	1 [Reference]	250/564 (44.5)	1 [Reference]
Employed	153/251 (64.1)	1.80 (1.17-2.77)	52/236 (23.0)	2.34 (1.23-4.47)	255/458 (59.3)	2.06 (1.46-2.91)
Not employed	144/341 (49.7)	1 [Reference]	46/398 (11.3)	1 [Reference]	216/510 (41.4)	1 [Reference]
Income below poverty guidelines	240/487 (54.7)	0.86 (0.52-1.41)	79/508 (15.0)	0.83 (0.43-1.61)	351/735 (49.0)	0.87 (0.61-1.25)
Income above poverty guidelines	57/105 (58.5)	1 [Reference]	19/126 (17.4)	1 [Reference]	120/233 (52.5)	1 [Reference]
Receiving Medicaid^c^	17/74 (26.8)	0.22 (0.11-0.44)	3/93 (3.7)	0.13 (0.04-0.43)	33/100 (30.1)	0.33 (0.20-0.57)
Receiving Medicare^d^	28/82 (45.6)	0.51 (0.26-0.99)	6/111 (7.6)	0.28 (0.07-1.05)	32/109 (38.2)	0.48 (0.23-0.99)
Private insurance	42/66 (67.5)	1.26 (0.60-2.66)	11/74 (16.3)	0.65 (0.30-1.45)	63/133 (47.7)	0.70 (0.42-1.17)
Uninsured	210/373 (62.3)	1 [Reference]	78/355 (23.0)	1 [Reference]	343/626 (56.5)	1 [Reference]

Abbreviations: OR, odds ratio; CI, confidence interval.

^a^ Percentages use weighted data.

^b^ ORs are for not self-reporting diagnosis for each variable.

^c^ Medicaid refers to Medicaid or Medicaid managed care.

^d^ Medicare refers to Medicare or Medicare managed care.

**Table 3:** Univariable Analysis of Weighted Data for Continuous Variables for Treatment, Cameron County Hispanic Cohort (N = 2,000), 2000–2008

Variable	Using Medication	Diabetes (n = 593)		Hypertension (n = 633)		Hypercholesterolemia (n = 958)	

		Mean (SE)	Mean Difference (95% CI)	Mean (SE)	Mean Difference (95% CI)	Mean (SE)	Mean Difference (95% CI)
Age in years	Yes	57.9 (1.1)	8.7 (4.9 to 12.4)	64.8 (1.0)	12.8 (10.1 to 15.6)	63.1 (1.3)	14.1 (11.0 to 17.2)
	No	49.2 (1.7)		52.0 (1.3)		49.0 (1.0)	
Household size	Yes	3.5 (0.2)	−0.1 (−0.4 to 0.3)	3.0 (0.1)	−0.6 (−0.9 to −0.2)	2.9 (0.2)	−0.8 (−1.2 to −0.3)
	No	3.5 (0.1)		3.5 (0.1)		3.7 (0.1)	
Higher level of education	Yes	7.6 (0.3)	−2.0 (−2.9 to −1.1)	7.9 (0.4)	−1.2 (−2.1 to −0.3)	7.3 (0.5)	−2.6 (−3.6 to −1.6)
	No	9.6 (0.3)		9.0 (0.3)		10.0 (0.2)	
MMSE Spanish score^a^	Yes	30.8 (1.6)	−1.3 (−5.9 to 3.3)	33.1 (1.3)	2.0 (−2.8 to 6.7)	30.9 (2.2)	−2.8 (−7.6 to 2.0)
	No	32.1 (1.7)		31.1 (1.8)		33.7 (1.0)	
MMSE English score^a^	Yes	17.0 (1.6)	−8.2 (−13.0 to −3.5)	18.9 (1.7)	−4.1 (−8.7 to 0.4)	16.6 (2.3)	−8.8 (−13.7 to −4.0)
	No	25.2 (1.7)		23.0 (1.6)		25.4 (1.0)	
Years resident in Brownsville	Yes	30.9 (1.4)	3.9 (−0.4 to 8.2)	36.3 (1.9)	10.6 (6.7 to 14.5)	36.2 (2.1)	11.5 (6.6 to 12.4)
	No	27.0 (1.8)		25.7 (1.9)		24.7 (1.4)	

Abbreviations: MMSE, Mini-Mental State Examination; SE, standard error; CI, confidence interval.

^a^ English and Spanish literacies are assessed using the language panels from the MMSE package (14).

**Table 4:** Univariable Analysis of Weighted Data for Categorical Variables for Treatment, Cameron County Hispanic Cohort (N = 2,000), 2000–2008

Variable	Diabetes (n = 593		Hypertension (n = 633)		Hypercholesterolemia (n = 958)	

	Not on Medication, n/total (%)^a^	OR (95% CI)^b^	Not on Medication, n/total (%)^a^	OR (95% CI)^b^	Not on Medication, n/total (%)^a^	OR (95% CI)^b^
Male	111/200 (60.2)	1.00 (0.65-1.52)	98/203 (39.4)	0.73 (0.50-1.06)	305/354 (84.2)	1.01 (0.64-1.60)
Female	220/393 (60.3)	1 [Reference]	219/431 (47.2)	1 [Reference]	519/614 (84.1)	1 [Reference]
Born in Mexico	231/405 (59.4)	0.92 (0.59-1.45)	204/409 (44.0)	1.00 (0.68-1.48)	563/656 (84.3)	1.00 (0.61-1.62)
Born in USA	97/184 (61.4)	1 [Reference]	108/216 (43.9)	1 [Reference]	253/300 (84.3)	1 [Reference]
Graduated from high school	126/191 (73.6)	2.46 (1.55-3.90)	117/197 (53.4)	1.74 (1.17-2.59)	461/564 (79.4)	2.67 (1.64-4.35)
Not graduated from high school	205/401 (53.2)	1 [Reference]	199/435 (39.8)	1 [Reference]	363/403 (91.2)	1 [Reference]
Employed	164/251 (68.8)	1.82 (1.19-2.81)	149/236 (61.0)	2.92 (1.81-4.71)	431/458 (94.8)	6.17 (3.49-10.91)
Not employed	167/342 (54.8)	1 [Reference]	168/398 (34.9)	1 [Reference]	393/510 (74.8)	1 [Reference]
Income below poverty guidelines	271/488 (60.0)	0.95 (0.58-1.60)	240/508 (40.9)	0.48 (0.30-0.78)	611/735 (82.2)	0.44 (0.24-0.82)
Income above poverty guidelines	60/105 (61.2)	1 [Reference]	77/126 (59.1)	1 [Reference]	213/233 (91.3)	1 [Reference]
Receiving Medicaid^c^	21/72 (31.6)	0.22 (0.12-0.42)	21/93 (18.8)	0.15 (0.08-0.30)	64/100 (57.3)	0.12 (0.06-0.22)
Receiving Medicare^d^	34/82 (50.9)	0.50 (0.27-0.94)	30/111 (22.5)	0.19 (0.11-0.35)	66/109 (65.9)	0.17 (0.09-0.33)
Private insurance	44/66 (70.9)	1.18 (0.56-2.49)	43/74 (58.4)	0.93 (0.49-1.74)	121/133 (91.5)	0.94 (0.41-2.18)
Uninsured	232/373 (67.4)	1 [Reference]	223/355 (60.3)	1 [Reference]	573/626 (92.0)	1 [Reference]

Abbreviations: CI, confidence interval; OR, odds ratio.

^a^ Percentages use weighted data.

^b^ Odds ratios are for not receiving appropriate medication for the condition for each variable.

^c^ Medicaid refers to Medicaid or Medicaid Managed Care.

^d^ Medicare refers to Medicare or Medicare Managed Care.

**Table 5:** Multivariable Analysis Using Weighted Data of Factors Associated With Being Undiagnosed and Not on Medication for Diabetes, Hypertension, and Hypercholesterolemia, Cameron County Hispanic Cohort (N = 2,000), 2000–2008

Variable	Diabetes (n = 593)	Hypertension (n = 633)	Hypercholesterolemia (n = 958)

	OR^a^ (95% CI)	OR^a^ (95% CI)	OR^a^ (95% CI)
Likelihood of not having condition diagnosed			
Older age^b^	0.98 (0.96-0.99)	1.01 (0.98-1.03)	0.98 (0.97-0.99)
Sex (male vs female)	0.79 (0.51-1.23)	1.26 (0.67-2.38)	0.90 (0.61-1.33)
Higher level of education^b^	1.79 (1.08-2.97)	1.85 (0.93-3.69)	1.42 (0.92-2.19)
Employed (vs other employment status)^b^	1.20 (0.76-1.90)	NA	1.80 (1.17-2.77)
More years in Brownsville^b^	NA	NA	1.01 (0.99-1.07)
Receiving Medicaid (vs not receiving Medicaid)	0.37 (0.17-0.77)	0.12 (0.04-0.44)	0.54 (0.30-0.96)
Receiving Medicare (vs not receiving Medicare)	1.21 (0.54-2.68)	0.23 (0.07-0.76)	0.92 (0.45-1.86)
Have private insurance (vs not having private insurance)	0.97 (0.47-2.01)	0.51 (0.22-1.17)	0.51 (0.29-0.99)
Likelihood of not being on medication			
Older age^b^	0.98 (0.96-0.99)	0.95 (0.93-0.97)	0.97 (0.95-0.99)
Sex (male vs female)	0.85 (0.55-1.33)	0.67 (0.43-1.02)	0.75 (0.44-1.29)
Higher level of education^b^	2.06 (1.24-3.43)	NA	1.75 (0.95-3.21)
Employed (vs other employment status)^b^	NA	NA	3.43 (1.83-6.44)
Receiving Medicaid (vs not receiving Medicaid)	0.35 (0.17-0.71)	0.34 (0.16-0.72)	0.30 (0.14-0.61)
Receiving Medicare (vs not receiving Medicare)	1.10 (0.52-2.30)	0.57 (0.28-1.16)	0.60 (0.26-1.41)
Have private insurance (vs not receiving private insurance)	0.89 (0.43-1.87)	0.92 (0.49-1.74)	0.59 (0.23-1.48)

Abbreviations: OR, odds ratio; CI, confidence interval; NA, not applicable.

^a^ Odd ratios are for not having the condition diagnosed.

^b^ Continuous variable.

Education in univariable analyses reduced the likelihood of both diagnosis (Table 1 and Table 2) and treatment (Table 3 and Table 4) of all 3 diseases, but our model (Table 5) confirmed this association only for diabetes. Participants who were employed were significantly less likely to self-report all 3 conditions and to be treated for diabetes and hypercholesterolemia; our model confirmed this effect only for hypercholesterolemia. In both univariable and multivariable analyses, the likelihood of receiving diagnosis and treatment for all 3 diseases was highest for participants who had insurance coverage. All participants with undiagnosed conditions were untreated. More than half of participants with diabetes (55.8%) were untreated among whom 29.9% had insurance and 10% had their diabetes diagnosed (data not shown). Of the drugs being taken for diabetes, 28% appeared to be of Mexican origin, many of which are not available in the United States. Half of participants with hypertension (50.0%) were untreated among whom 30% had insurance and 69.1% had their hypertension diagnosed. Taking lipid-lowering drugs was uncommon. Most participants with elevated cholesterol did not receive treatment (85.1%), among whom 30.7% were insured, and 42.8% had their hypercholesterolemia diagnosed. Few participants with high cholesterol (8.9%) took expensive drugs, such as statins, and only 3.8% reported taking preventive over-the-counter supplements, such as omega-3 fatty acids.

Lack of insurance affected several measurements. Participants with diabetes who had insurance had a mean A1c of 7.9% (±0.16), but participants without insurance had a higher mean level, 8.6% (±0.21, *P* = .005). Overall, the prevalence of other biomarkers for chronic diseases was high. Mean C-reactive protein in participants with diabetes was 5.8 mg/L.

The associations with insurance and employment were complex. Only 28.5% of participants reported having health insurance of any kind, and half of these received Medicare or Medicaid. As expected, the highest rates of health insurance (87%) were among those aged 65 years or older, most of whom were on Medicare. Participants without private insurance were shown in a logistic regression model to be younger, have lower incomes, come from larger families, have been born in Mexico, and have lived less time in Brownsville (data not shown). The strongest predictor in the model of not having insurance, however, was not having completed high school. Very few recipients of Medicare or Medicaid insurance were on managed care (15 of 160 on Medicare managed care and 13 of 158 on Medicaid managed care). Of the 651 participants employed full time, 435 (66.8%) had no health insurance; 300 (91.5%) of 328 participants employed part time had no health insurance.

## Discussion

We report data from a cohort of community-recruited Mexican Americans with health disparities showing widespread failure to diagnose and treat diabetes, hypertension, and hypercholesterolemia, which are most treatable in early stages. However, the reasons for failure to diagnose and treat these diseases are more complex than lack of access to care. If this failure is not addressed, it will lead to increasing socioeconomic and health care costs.

In our study population, the prevalence of all 3 diseases was higher than that reported in 2001 for the entire US population on the US-Mexico border (7). This is already in line with the prediction from the Centers for Disease Control and Prevention that 1 in 3 people in the United States will have diabetes by 2050 (18), at which time Hispanics are expected to constitute 30% of the US population. Treatment was dependent on diagnosis, but even participants with diagnosed diabetes, hypertension, or hypercholesterolemia often were not on medication, particularly those participants without insurance.

The typical participant with undiagnosed and untreated diabetes was younger, better educated, and had good English literacy. Younger participants were less likely to have had diabetes diagnosed or to be on medication. This may be because younger people do not perceive themselves at risk for diabetes. The high rate of diabetes treatment with medication in participants who had had diabetes diagnosed and had insurance suggests that these participants understood the importance of treatment and accessed treatment; however, the lower level of diagnosis and treatment of diabetes in the better educated participants was unexpected.

Participants self-reported hypertension more frequently than diabetes or hypercholesterolemia, but those participants with diagnosed hypertension who were untreated were younger than those who were treated. Two-thirds of participants with diagnosed hypertension who also had insurance were receiving treatment. This suggests that participants and local physicians understood the importance of treating hypertension.

Most participants with hypercholesterolemia were young, employed, and had not received diagnosis and treatment, suggesting that participants may be unaware of the benefits of treating the disease. In addition, the cost of the most effective cholesterol-lowering drugs, such as statins, could be prohibitive even for the 40% who were insured. We also found that almost none of our participant population used over-the-counter medications that lower cholesterol, such as omega-3 fatty acids. The consequences of failure to address these diseases can be seen in increased rates of poorly controlled diabetes.

In none of our models did we find that income below poverty guidelines had any effect on diagnosis or treatment. However, we did observe a very strong effect in people receiving Medicaid that favored both diagnosis and treatment of all 3 diseases. This finding is consistent with findings from our qualitative focus group studies indicating that people who have Medicaid can get treatment (B.M.R., unpublished data, May 2012). This encourages expectations that the Affordable Health Care Act will benefit a wide population (19). Changes being considered by the US Food and Drug Administration to make common medications for diabetes, hypertension, and hypercholesterolemia available without prescription would be helpful, freeing up clinics and streamlining processes for patients (20). Off-label statins are now available and more affordable (21).

Study participants on Medicare did not get clear benefits from insurance coverage except for the diagnosis of hypertension. Private insurance did not appear to benefit participants, again suggesting lack of awareness of the need for treatment among participants. Factors such as denial of illness and reluctance to seek care are confirmed by a report from France showing lack of diagnosis and treatment of diabetes in many people with access to medical care (22).

Our data concerning Medicaid does show that widespread lack of preventive medicine and appropriate medication is to some degree related to lack of health insurance. Hispanic populations have the highest proportion of people without medical insurance in the United States (32.7%) compared with non-Hispanic whites (10.7%), African Americans (19.4%), and the US population overall (15.3%) (23). Most of the uninsured were young, an age group that should be targeted with preventive health care. Failure to obtain insurance in adults aged 65 or younger is related to poverty, as is being a recent immigrant; recent immigrants are also likely to live in poverty (24). Employment as an influence on diagnosis and treatment is complex, because much employment in our study participants involved hourly wages without benefits but with income levels that made them ineligible for Medicaid.

We found that limited education was a stronger independent determinant of not having health insurance than poverty, immigration status, or employment status. Greater educational attainment in this population appears to promise improvement in accessing health benefits and the health care delivery system.

This study has several limitations. Not all participants with 1 or more of the 3 chronic conditions studied may have required medication; however, the high levels of A1c among participants with diabetes suggest that disease not requiring medication is uncommon. In our qualitative study we heard that people may stop taking medication when they feel better or because the medication makes them feel unwell (B.M.R., unpublished data). Several study participants who were not taking medication may have been treated with medication in the past.

The strengths of our study are that the data are from a large, randomly selected representative population and that it provides a view of the volume of undiagnosed and untreated chronic disease in a community with health disparities. This is a community where local ophthalmologists commonly make the primary diagnosis of diabetes in a patient who seeks care for failing vision and where the only access to diagnosis and treatment for many is a hospital emergency department. The high volume of undiagnosed and untreated diabetes, hypertension, and hypercholesterolemia results in a substantial burden of health care expenditure, economic loss, and premature mortality (24,25).

Our study casts light on a missed opportunity for reducing illness and death from common chronic diseases in a minority population. Addressing this missed opportunity would reduce long-term medical, social, and economic burdens. A large proportion of US health care dollars are spent on end-of-life care (26). We need a shift in public perception of values toward education and prevention in primary care settings (19). Our study provides a portrait of a young, highly disparate, largely uninsured minority population with extensive neglect of chronic disease. In October 2011, the World Economic Forum estimated that by 2030 chronic disease will cost $47 trillion globally (27). The economic toll of diabetes alone in the workplace in the Lower Rio Grande Valley, where this cohort resides, is estimated to be $227 million a year in lost wages (28). Preventive medicine is key to controlling the economic effect of chronic diseases in minority communities. Neglecting and ignoring disease trends in populations with health disparities will have costly consequences, not only for those populations but for the nation as a whole.
